# Cook County Hospital

**Published:** 1884-03

**Authors:** E. P. Davis


### Hospital Reports.

### ARTICLE VI.

COOK COUNTY HOSPITAL.

The meeting of the recently elected medical board resulted in the assignment of the following gentlemen for duty:

As surgeons, Dr. T. W. Miller; Dr. R. L. Rea; as physicians, Dr. S. A. McWilliams; Dr. P. H. Cronin; gynæcologist and obstetrician, Dr. John Guerin; oculist and aurist, Dr. L. D. Jacobson; pathologist, Dr. W. T. Belfield.

Clinics are given as follows:

Monday, 2 P. M.—Autopsies by Dr. Belfield.

Tuesday, 1:30 P. M.—Medical clinic by Dr. McWilliams.

Tuesday, 2:30 P. M.—Surgical clinic by Dr. Rea.

Friday, 1:30 P. M.—Medical clinic by Dr. Cronin.

Friday, 2:30 P. M.—Surgical clinic by Dr. Miller.

The new pavilions are being fitted for occupancy; it is expected that the western pavilion will be in part occupied by the obstetric ward during February.

It is estimated that each pavilion will accommodate 75 patients.

The upper floors of these buildings are divided into small rooms, available for private patients or cases needing special attention.

The new buildings will contribute a surgical, gynæcological, obstetric and medical ward to the capacity of the hospital—a much needed addition, as the wards now in use are continually overcrowded during the winter.

In finish and arrangement they are much superior to the wards now occupied. The central or official building will not be completed before the autumn; the estimated cost of the two pavilions and central building will be about $300,000.

The profession will be interested in knowing that the warden is devising an arrangement for tabulating the histories of patients; as formerly kept, the records furnish no scientific data.

E. P. DAVIS, M.D.

---

ALCOHOLIC PARALYSIS AND CEREBRAL SYPHILIS.

Alcoholic paralysis is more common among women than among men; it is symmetrical and localized to homologous muscular groups of both sides. The lower extremities are more affected than the upper. The extensor muscles are more attacked than the flexors. The paralytic troubles are accompanied by analgesia or symmetrical hyperalgesia, by convulsions, or the sensation of formication, or stitches in the ends of the limbs. There are also vaso-motor troubles, *i. e.*, redness or paleness of the skin, or œdema of the back of the hands or feet. The patients were also affected with other symptoms of chronic alcoholism, *i. e.*, gastric troubles, hallucinations, etc. The faradic current has but little effect on the paralyzed muscles. The patients die mostly from marasmus, coma, or a pulmonary trouble, and in the autopsy there is found an alteration in the nerves and muscles, while the medulla and spinal cord are healthy.

PROLONGED DIPHTHERIA.

A young physician performing tracheotomy was infected with diphtheria of the nasal cavities and the throat. For nine days all the symptoms of diphtheria were present, but after that time he furnished for nine months pseudo-membranes which had the exact form of the nasal cavities. In another case of a little boy 2 years old, where tracheotomy had been performed, the pseudo-membranes from the trachea were cast off for 154 days, and in another case the membranes appeared for 43 days per tracheam. —*Jour. des Maladies des femmes.*

